# Role of Dietary Nutritional Treatment on Hepatic and Intestinal Damage in Transplantation with Steatotic and Non-Steatotic Liver Grafts from Brain Dead Donors

**DOI:** 10.3390/nu13082554

**Published:** 2021-07-26

**Authors:** Marc Micó-Carnero, Araní Casillas-Ramírez, Albert Caballeria-Casals, Carlos Rojano-Alfonso, Alfredo Sánchez-González, Carmen Peralta

**Affiliations:** 1 Institut d’Investigacions Biomèdiques August Pi i Sunyer (IDIBAPS), 08036 Barcelona, Spain; mico@clinic.cat (M.M.-C.); acabalca31@alumnes.ub.edu (A.C.-C.); rojano@clinic.cat (C.R.-A.); 2 Hospital Regional de Alta Especialidad de Ciudad Victoria “Bicentenario 2010”, 87087 Ciudad Victoria, Mexico; aranyc@yahoo.com (A.C.-R.); asg_4@live.com (A.S.-G.); 3 Facultad de Medicina e Ingeniería en Sistemas Computacionales de Matamoros, Universidad Autónoma de Tamaulipas, 87300 Matamoros, Mexico

**Keywords:** brain death, liver transplantation, steatotic liver grafts, ischemia-reperfusion, polysaccharides, glucose, lipid emulsion, intestinal inflammation, gut microbiota

## Abstract

Herein, we investigate whether: (1) the administration of glucose or a lipid emulsion is useful in liver transplantation (LT) using steatotic (induced genetically or nutritionally) or non-steatotic livers from donors after brain death (DBDs); and (2) any such benefits are due to reductions in intestinal damage and consequently to gut microbiota preservation. In recipients from DBDs, we show increased hepatic damage and failure in the maintenance of ATP, glycogen, phospholipid and growth factor (HGF, IGF1 and VEGFA) levels, compared to recipients from non-DBDs. In recipients of non-steatotic grafts from DBDs, the administration of glucose or lipids did not protect against hepatic damage. This was associated with unchanged ATP, glycogen, phospholipid and growth factor levels. However, the administration of lipids in steatotic grafts from DBDs protected against damage and ATP and glycogen drop and increased phospholipid levels. This was associated with increases in growth factors. In all recipients from DBDs, intestinal inflammation and damage (evaluated by LPS, vascular permeability, mucosal damage, TLR4, TNF, IL1, IL-10, MPO, MDA and edema formation) was not shown. In such cases, potential changes in gut microbiota would not be relevant since neither inflammation nor damage was evidenced in the intestine following LT in any of the groups evaluated. In conclusion, lipid treatment is the preferable nutritional support to protect against hepatic damage in steatotic LT from DBDs; the benefits were independent of alterations in the recipient intestine.

## 1. Introduction

At present, some 80% of grafts are taken from donors after brain death (DBDs). However, brain death (BD) markedly reduces tolerance of preservation/reperfusion injury among liver grafts and reduces graft survival [1,2]. In clinical liver transplantation (LT), the shortage of hepatic graft donors, and consequently the increase in waiting lists for LT, has led centers to relax their criteria for the acceptance of organs from marginal donors, such as steatotic liver grafts. Up to 50% of deceased donor livers are estimated to be steatotic and steatosis is recognized to be a key donor variable when it comes to predicting post-transplant outcomes [3]. A further increase in the prevalence of steatosis in society in general and therefore also in deceased donor livers is expected. It is important to note that hepatic steatosis represents a greater risk of organ dysfunction and primary non-function when compared to non-steatotic livers [2]. Furthermore, many steatotic livers, especially those with severe fatty infiltration, are excluded from consideration for LT, which exacerbates the critical shortage of liver donors [1]. Progress in this area would reduce the inherent risk of dysfunction or failure after LT and would also help to shorten LT waiting lists.

Hepatic ischemia-reperfusion (I/R) associated with LT may induce pathological intestinal alterations and multiple organ failure [4]. This is due to intestinal congestion in the recipient during the anhepatic phase of implantation of liver grafts and the release of pro-inflammatory mediators from the damaged liver into circulation during reperfusion [5]. Evidence indicates that this latter process is mediated by neutrophil infiltration [6]. Moreover, microvascular dysfunction, coupled with the release of inflammatory mediators including reactive oxygen species, tumor necrosis factor-1α (TNF-1α) and interleukin-1 (IL1), may induce tissue injury, which can also lead to organ dysfunction and failure [7]. In the light of all this, a large body of accumulated research evidence indicates that the modulation of intestinal inflammation and damage resulting from hepatic I/R has a positive effect on the quality of liver grafts and post-operative outcomes after LT [8,9,10].

It has been convincingly demonstrated that disbalances of the intestinal microbiota can potentiate proinflammatory pathways, which results in intestinal inflammation and damage [11,12]. Meanwhile, it is known that polysaccharides are the most abundant element in typical diets [13]. Moreover, polysaccharides can affect consumer health by modulating the gut microbiome, which plays crucial roles in numerous digestive disorders such as inflammatory bowel diseases or different liver pathologies [13]. However, it is not only polysaccharides that are important in modulation of the gut microbiome; it is known that glucose and various lipids can influence gut microbial diversity and consequently affect gut health [14].

Previous results from studies of partial hepatectomy (PH) under ischemia-reperfusion (I/R) indicate that lipid treatment in non-steatotic livers provides the same protection as that afforded by glucose. Meanwhile, in the presence of steatosis, treatment with lipids is the preferable option to reduce the injurious effects of liver surgery [15,16]. Taken together, these data concerning the effects of treatment based on lipids/glucose and those mentioned above on the effect of polysaccharides/glucose and lipids on gut health [16,17] support the hypothesis that nutritional support (treatment with either glucose or a lipid emulsion) could not only be effective against local (liver) I/R induced injury, but could also attenuate damage in remote organs such in the intestine, by modulating the mechanisms involved in the pathogenesis of intestinal inflammation. If this were indeed the case, such nutritional support could protect liver grafts both directly and indirectly, through its beneficial effects on the intestinal inflammation resulting from hepatic I/R.

Given all these observations and the close relationship between the gut and liver, we cannot rule out the possibility that intestinal damage and dysbiosis in recipients that are a result of LT can compromise liver graft viability and consequently reduce liver function in LT recipients from DBDs. In the light of this, we aim here to resolve this issue and to elucidate: (a) the potential benefits that result from the administration of either glucose or lipids in steatotic and non-steatotic LT from DBDs; and (b) whether these effects can be explained by potential improvements in intestinal inflammation and damage. Thus, if we observe potential improvements in intestinal inflammation and damage, we will next go on to study whether these are caused by intestinal dysbiosis. In this way our principal idea is to elucidate the as yet unidentified pathophysiological mechanisms in steatotic and non-steatotic LT from DBDs and thereby to identify novel strategies that could be applied in LT from DBDs; all within a setting in which more than 80% of transplants are from cadaveric donors and more than 50% of donors present hepatic steatosis [18,19].

## 2. Materials and Methods

### 2.1. Experimental Animals

We used male homozygous, obese (Ob) (350–400 g) and heterozygous, lean (Ln) (400–450 g) Zucker rats as our genetic obesity experimental model; and male Wistar rats (200–220 g) fed with a choline-deficient or standard chow diet for 10 days [20] as our nutritional obesity experimental model. Ob Zucker and Ob Wistar rats showed severe macrovesicular and microvesicular fatty infiltration in hepatocytes (60–70% steatosis) [21]. The chow for Zucker rats and non-obese Wistar rats was composed with 66% of polysaccharides (Teklad Global 14% Protein Rodent Maintenance Diet, ENVIGO), whereas choline-deficient diet fed Wistar rat chow was composed of 25% of polysaccharides (Dyets, Inc. Bethlem, PA, USA). This will provide information about the role of potential differences in the context of dietary polysaccharides of animals on gut damage and inflammation. All procedures were conducted according to European Union regulations for animal experiments (Directive 86/609 EEC).

### 2.2. Experimental Design

To study the effects of lipid and glucose administration on hepatic damage and intestinal inflammation in LT from DBDs in our genetic obesity experimental model, we established the following experimental groups.

1. Sham (*n* = 12, 6 Ln and 6 Ob): Ob and Ln Zucker rats were anesthetized, ventilated and maintained as normotensive with saline infusion for 6 h [21,22].

2. LT (*n* = 24, 12 transplantations: 6 with non-steatotic grafts and 6 with steatotic grafts): Ob and Ln Zucker rats were anesthetized, ventilated and maintained as normotensive with saline infusion for 6 h. Then, steatotic and non-steatotic livers were flushed with University of Wisconsin (UW) solution, isolated, preserved in ice-cold UW solution for 4 h and implanted into Ln Zucker rats [21,22,23].

3. BD + LT (*n* = 24, 12 transplantations: 6 with non-steatotic grafts and 6 with steatotic grafts): Ob and Ln Zucker rats were anesthetized and ventilated. To induce BD, a frontolateral trepanation was performed on the rats and a balloon catheter was introduced in the extradural space. The intracranial pressure was increased by inflating the balloon for one minute to induce rapid brain injury, leading to immediate BD [24]. The rats were then maintained normotensive with a colloid infusion for 6 h. After this, livers were flushed with UW solution, isolated, preserved in ice-cold UW solution for 4 h and implanted into Ln Zucker rats [21,22].

4. BD + LT + Glucose (*n* = 24, 12 transplantations: 6 with non-steatotic grafts and 6 with steatotic grafts): The same as group 3, except that the recipients were intravenously infused for 4 h with 5 mL of a glucose solution (28%, energy content 4.6 MJ/1000 mL, Sigma Aldrich, Spain) immediately after the implantation of the liver graft [25].

5. BD + LT + Lipid (*n* = 24, 12 transplantations: 6 with non-steatotic grafts and 6 with steatotic grafts): The same as group 3, except that the recipients were intravenously infused for 4 h with 5 mL of a lipid solution (10% Intralipid; 4.6 MJ/1000 mL, Fresenius Kabi, Barcelona, Spain) immediately after the implantation of liver graft [25]. The emulsion was comprised of 52% linoleic acid, 22% oleic acid, 13% palmitic acid, 8% linolenic acid, 4% stearic acid, 1% other fatty acids, 8.184 g/L egg phospholipids and 15 g/L glycerin [25,26].

To evaluate the effects of lipid and glucose administration on hepatic damage and intestinal inflammation in LT from DBDs in the nutritionally obesity experimental model, we performed the same surgical procedures under the same conditions as those described for groups 1–5, but using Wistar rats fed the choline-deficient diet (Ob Wistar rats) instead of Ob Zucker rats, and Wistar rats fed the standard chow diet (Ln Wistar rats) instead of Ln Zucker rats.

Samples were collected 4 h after LT. The conditions of this study (including the doses and pre-treatment times used for the different drugs) were selected on the basis of previous studies reported above and preliminary studies by our group [25,26,27]. A cold ischemic period of 4 h is long enough to induce liver damage after LT in liver grafts and allows a high rate of survival at 4 h after LT. In fact, we also carried out survival studies and observed that the survival rate was drastically reduced at 6 h after LT, especially in recipients with steatotic liver grafts (all recipients with steatotic livers died by this time point). Thus, the experimental conditions used in the current study were the most appropriate for our aim, that is to say, to evaluate the effect of either glucose or lipids on hepatic damage and intestinal inflammation in LT from DBDs as well as the potential changes in gut microbiota if improvements in intestinal alterations following LT were observed when the dietary supplements were administered.

### 2.3. Biochemical Determinations

We determined levels of tansaminases, alkaline phosphatase (ALP), bilirubin, hyaluronic acid (HA), von Willebrand factor (vWF), ATP, glycogen, HGF, IGF-1, VEGFA, LPS, TLR4, TNFα, IL1β and IL10, as described elsewhere [20,28,29,30,31]. Lipid peroxidation was determined by measuring the formation of malondialdehyde (MDA) using the thiobarbiturate reaction [32]. We photometrically determined myeloperoxidase (MPO), as an index of neutrophil accumulation, using 3,3′,5,5′-tetramethylbenzidine as a substrate [33]. Vascular permeability was estimated using the Evans Blue method [34]. Edema formation was measured by analyzing the wet-to-dry weight ratio after samples were heated to 55 °C [35]. Free fatty acids (FFA), triglycerides, total cholesterol and phospholipids were measured following standard procedures.

### 2.4. Histology

The severity of hepatic injuries was established using a point-counting method on an ordinal scale as follows: grade 0, minimal or no evidence of injury; grade 1, mild injury consisting of cytoplasmic vacuolation and focal nuclear pyknosis; grade 2, moderate to severe injury with extensive nuclear pyknosis, cytoplasmic hypereosinophilia, and loss of intercellular borders; grade 3, severe necrosis with disintegration of hepatic cords, hemorrhage, and neutrophil infiltration; and grade 4, very severe necrosis with disintegration of hepatic cords, hemorrhaging, and neutrophil infiltration [36]. Liver steatosis was evaluated via red oil staining. Freeze tissue in OCT compound were cut at 5 µm before being mounted on slides and dried. The sections were fixed in 10% formalin. Then, the samples were placed in 100% propylene glycol and stained in a 0.5% Red Oil solution in propylene glycol for 30 min. Finally, the slides were transferred to a solution of 85% of propylene glycol for 1 min before being stained with hematoxylin [37]. The grade of severity of steatosis was performed according the three different categories: mild (<30% of the hepatocytes with lipid droplets), moderate (30–60% of the hepatocytes with lipid droplets) and severe (>60% of the hepatocytes with lipid droplets) [38]. Intestinal mucosal damage was evaluated using Chiu’s score, [39] as follows: grade 0, normal mucosa villi; grade 1, development of subepithelial Gruenhagen’s spaces at the tip of the villi, with capillary congestion; grade 2, extension of the subepithelial space with moderate epithelial lifting; grade 3, massive epithelial lifting, possibly with a few denuded villi; grade 4, denuded villi with lamina propria and dilated capillaries exposed; grade 5, digestion and disintegration of the lamina propria, ulceration and hemorrhage.

### 2.5. Statistics

The statistical significance of differing variables was determined via the non-parametric Kruskal–Wallis test. The Mann–Whitney U test was applied to groups showing significant differences and adjusted *p*-values were calculated using the false discovery rate (FDR) method (we considered *p* < 0.05 to be significant).

## 3. Results

### 3.1. Effects of Lipid and Glucose Administration on Hepatic Damage in Steatotic and Non-Steatotic LT in a Genetic Obesity Experimental Model

**Figure 1:**
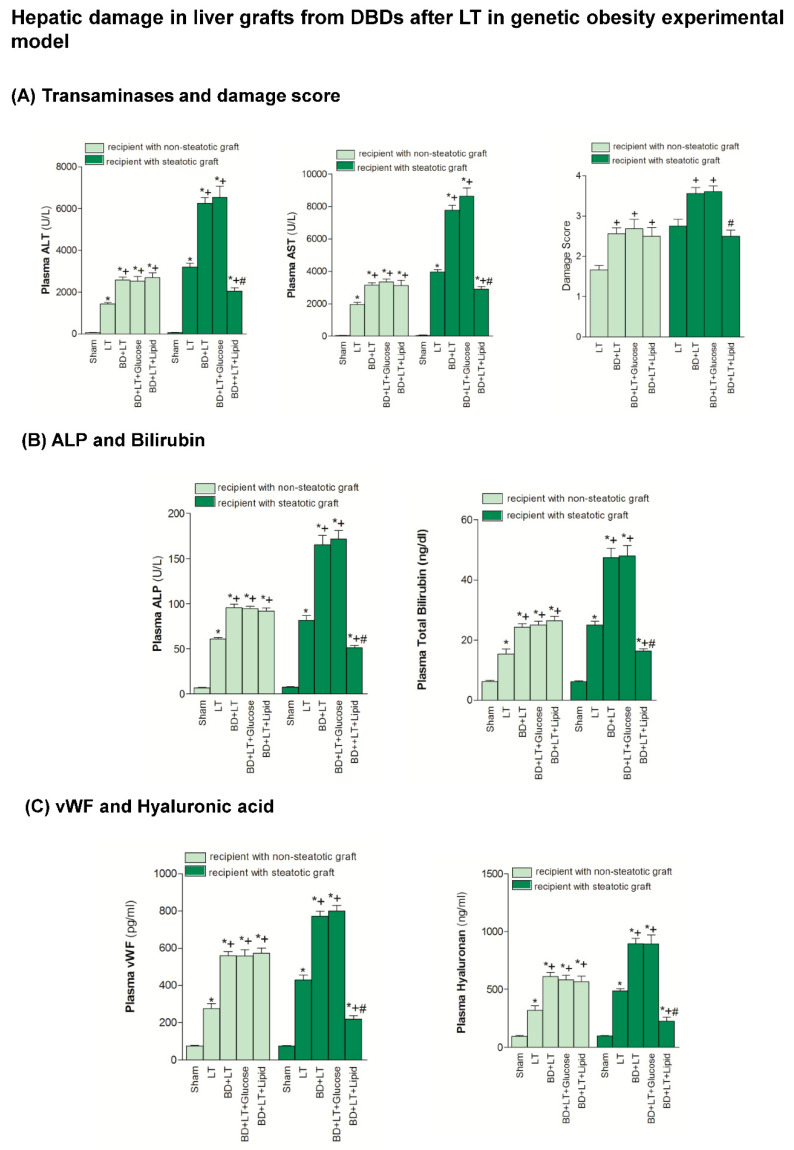
Effects of glucose and lipids on hepatic damage in steatotic and non-steatotic Liver transplantation (LT) in a genetic obesity experimental model. (**A**) AST and ALT in plasma, and damage score in liver. (**B**) alkaline phosphatase (ALP) and bilirubin in plasma. (**C**) von Willebrand factor (vWF) and hyaluronic acid in plasma. * *p* < 0.05 vs. Sham. + *p* < 0.05 vs. LT. # *p* < 0.05 vs. Brian death +Liver Transplantation (BD + LT). DBD, Donors after Brain death.

The BD + LT group resulted in exacerbated hepatic damage (transaminase, ALP and bilirubin) and endothelial cell damage, measured by vWF and HA levels, in both steatotic and non-steatotic grafts, when compared with the results of the LT group. We therefore studied the relevance of the treatment with lipids or glucose for hepatic damage in non-steatotic and steatotic LT from DBDs. We found that the administration of glucose (BD + LT + Glucose group) did not induce any change in the hepatic injury in either type of liver grafts, since it did not modify the transaminase, ALP, bilirubin, vWF or HA levels, compared to the BD + LT group (Figure 1). However, the administration of lipids (BD + LT + Lipid group) did protect against hepatic damage, but only in the presence of steatosis. This finding was reflected in a reduction of the transaminase, ALP, bilirubin, vWF and HA levels, compared to the results of the BD + LT group. In the BD + LT, BD + LT + Glucose and BD + LT + Lipid groups, our histological evaluation of non-steatotic livers showed moderate multifocal areas of coagulative necrosis and neutrophil infiltration, randomly distributed throughout the parenchyma. Meanwhile, in the steatotic grafts in the BD + LT and BD + LT + Glucose groups, we observed severe, extensive, and confluent areas of coagulative necrosis. For the BD + LT + Lipid group, we found reduced extensions and numbers of necrotic areas in both steatotic and non-steatotic livers. These histological results were evident in the values of the corresponding damage score (Figure 1).

### 3.2. Mechanisms Underlying Glucose and Lipid Action on Steatotic and Non-Steatotic Livers in a Genetic Obesity Experimental Model

**Figure 2:**
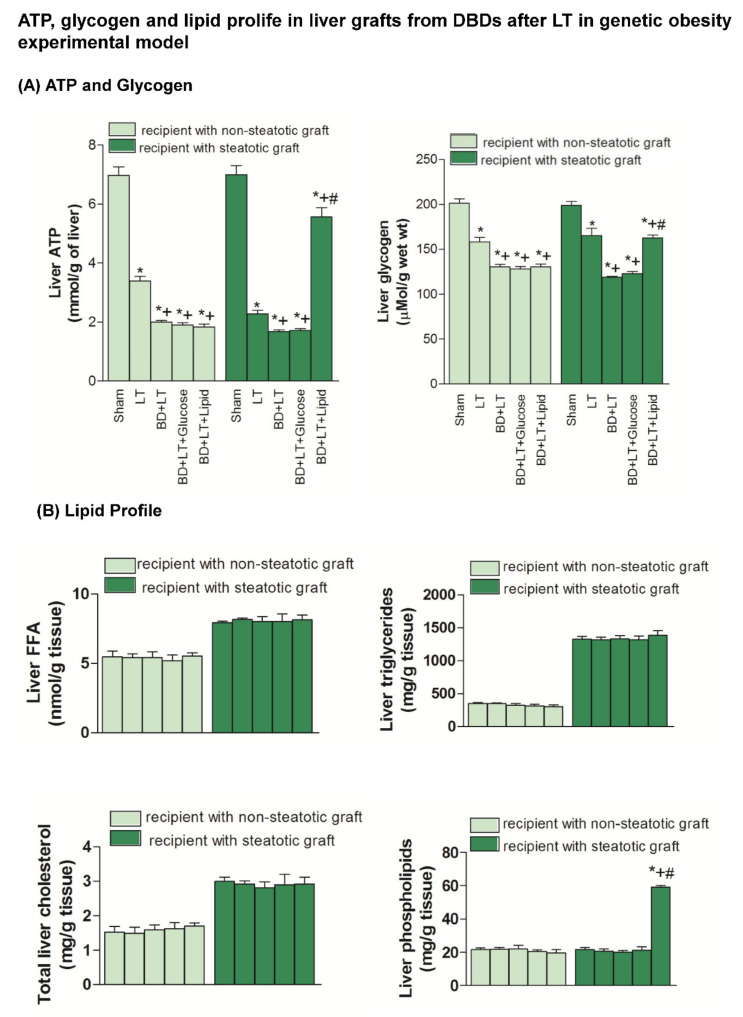
Effects of glucose and lipids on ATP, glycogen and lipid profile in steatotic and non-steatotic Liver Transplantation (LT) in a genetic obesity experimental model. (**A**) ATP and glycogen content in liver. (**B**) FFA, triglycerides, total cholesterol and phospholipids in liver. * *p* < 0.05 vs. Sham. + *p* < 0.05 vs. Liver Transplantation (LT). # *p* < 0.05 vs. Brain Death+Liver Transplantation (BD + LT). DBD, Donors after Brain death.

ATP and glycogen levels in the non-steatotic livers from the BD + LT + Glucose and BD + LT + Lipid groups were similar to those from the BD + LT group (Figure 2). No differences in hepatic ATP and glycogen levels were observed in steatotic livers, compared to those of the BD + LT + Glucose and BD + LT groups. However, the ATP and glycogen levels in the steatotic livers from the BD + LT + Lipid group were higher than those from the BD + LT group. Meanwhile, the FFA, triglycerides and total cholesterol levels were similar in all the groups. In the BD + LT + Lipid group, we observed increased phospholipid levels only in the steatotic livers when compared with the BD + LT group (Figure 2).

**Figure 3:**
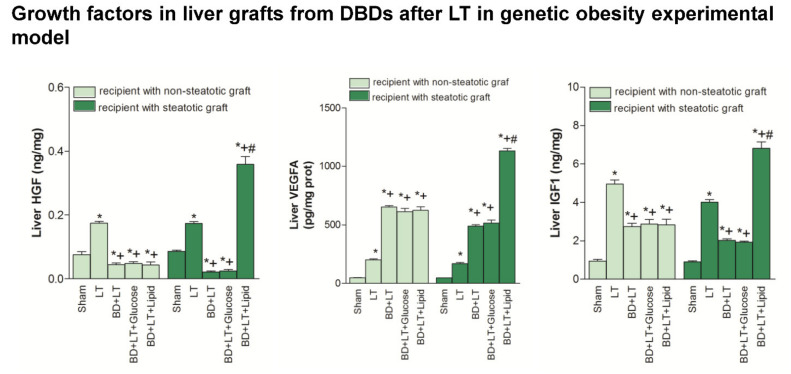
Effects of glucose and lipids on growth factors in steatotic and non-steatotic LT in a genetic obesity experimental model. HGF, VEGFA and IGF1 in liver. * *p* < 0.05 vs. Sham. + *p* < 0.05 vs. Liver Transplantation (LT). # *p* < 0.05 vs. Bran Death+Liver Transplantation (BD + LT). DBD, Donors after Brain death.

Next, we evaluated whether, in addition to the benefits in terms of APT, glycogen and phospholipids induced by the lipid treatment in steatotic livers, changes in growth factors (HGF, IGF1 and VEGFA) could explain the enhanced benefits conferred by the lipid treatment in the presence of steatosis. Our results showed that HGF, IGF1 and VEGFA levels in non-steatotic livers were similar in the BD + LT, BD + LT + Glucose and BD + LT + Lipid groups (Figure 3). Similarly, the HGF, IGF1 and VEGFA levels in steatotic livers from the BD + LT + Glucose group were similar to those from the BD + LT group. However, the steatotic livers from the BD + LT + Lipid group had higher HGF, IGF1 and VEGFA levels than those in the BD + LT group.

### 3.3. Role of Intestine in the Effects of Lipid and Glucose Administration on Hepatic Damage in Steatotic and Non-Steatotic LT from DBDs in a Genetic Obesity Experimental Model

**Figure 4:**
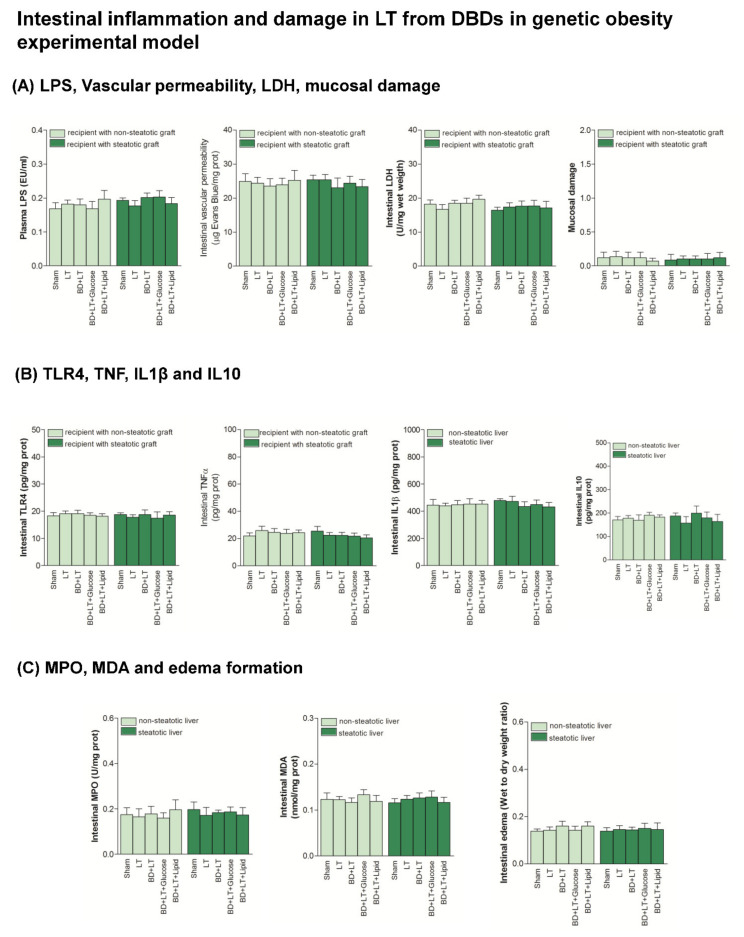
Effects of glucose and lipids on intestinal inflammation and damage in steatotic and non-steatotic LT in a genetic obesity experimental model. (**A**) LPS in plasma, and vascular permeability, LDH and mucosal damage in intestine. (**B**) Toll like Receptor 4 (TLR4), Tumor necrosis factor (TNF), Interleukin (IL)1β and Interleukin (IL)10 in intestine. (**C**) Myeloperoxidase (MPO), Malondialdehyde (MDA) and edema formation in intestine. Sham vs. Liver Transplantation (LT) or Brain Death+Liver Transplantation (BD + LT), *p* = not significant; LT vs. Brain Death+Liver Transplantation (BD + LT), *p* = not significant. DBD, Donors after Brain death.

Next, we considered the possibility that the beneficial effect of lipids on hepatic damage might be related to an improvement in intestinal inflammation, but this was ruled out because all the parameters that reflect intestinal inflammation and damage (LPS, vascular permeability, LDH, mucosal damage, TLR4, TNFα, IL1β, IL10, MPO, MDA and edema formation) in the BD + LT + Lipid group were similar to those of the BD + LT group (Figure 4). Indeed, no differences in the parameters for intestinal inflammation and damage were observed when comparing the sham control, LT and BD + LT groups.

### 3.4. Hepatic Damage and Intestinal Inflammation in LT from DBDs in a Nutritionally Induced Obesity Model

**Figure 5:**
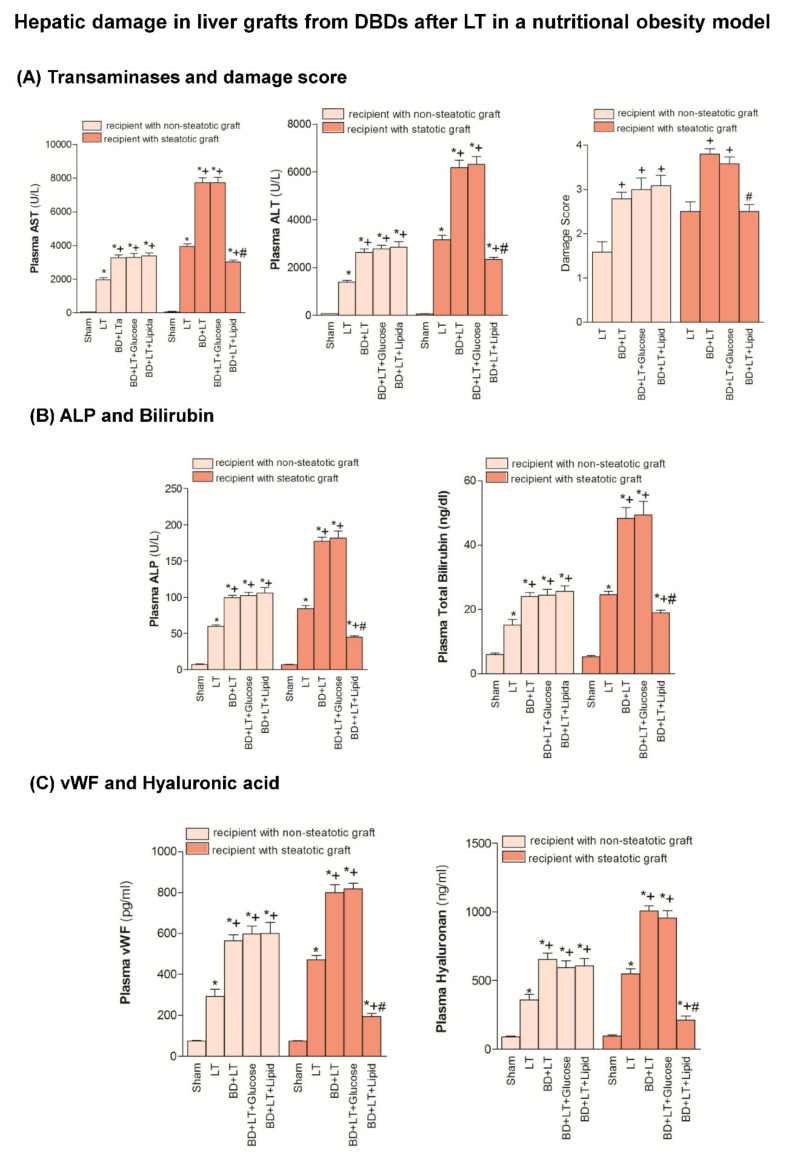
Effects of glucose and lipids on hepatic damage in steatotic and non-steatotic LT in a nutritional obesity model. (**A**) AST and ALT in plasma, and damage score in liver. (**B**) Alkaline phosphatase (ALP) and bilirubin in plasma. (**C**) von Willebrand factor (vWF) and hyaluronic acid in plasma. * *p* < 0.05 vs. Sham. + *p* < 0.05 vs. Liver Transplantation (LT). # *p* < 0.05 vs. Brain Death+Liver Transplantation (BD + LT). DBD, Donors after Brain death.

**Figure 6:**
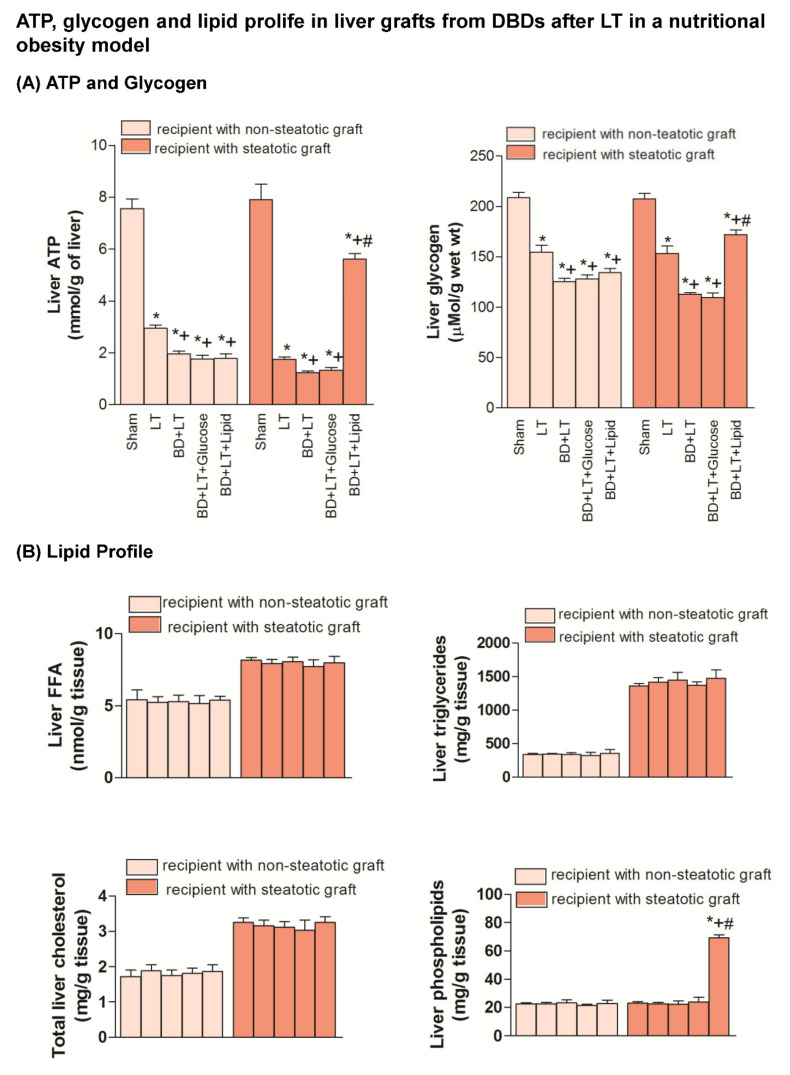
Effects of glucose and lipids on ATP, glycogen and lipid profile in steatotic and non-steatotic LT in a nutritional obesity model. (**A**) Adenosine triphosphate (ATP) and glycogen content in liver. (**B**) Free fatty acids (FFA), triglycerides, total cholesterol and phospholipids in liver. * *p* < 0.05 vs. Sham. + *p* < 0.05 vs. Liver Transplantation (LT). # *p* < 0.05 vs. Brain Death+Liver Transplantation (BD + LT). DBD, Donors after Brain death.

**Figure 7:**
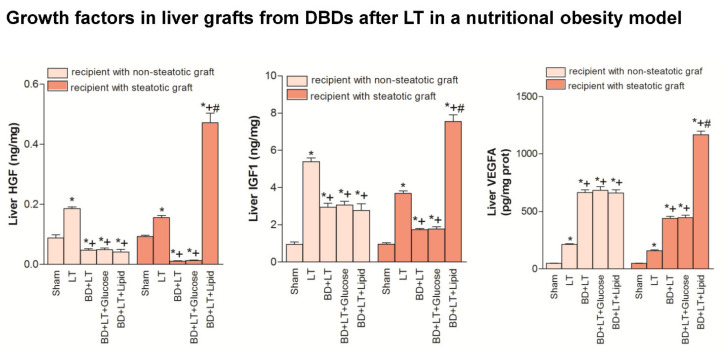
Effects of glucose and lipids on growth factors in steatotic and non-steatotic LT in a nutritional obesity model. HGF, IGF1 and VEGFA in liver. * *p* < 0.05 vs. Sham. + *p* < 0.05 vs. Liver Transplantation (LT). # *p* < 0.05 vs. Brain death+ Liver Transplantation (BD + LT). DBD, Donors after Brain death.

**Figure 8:**
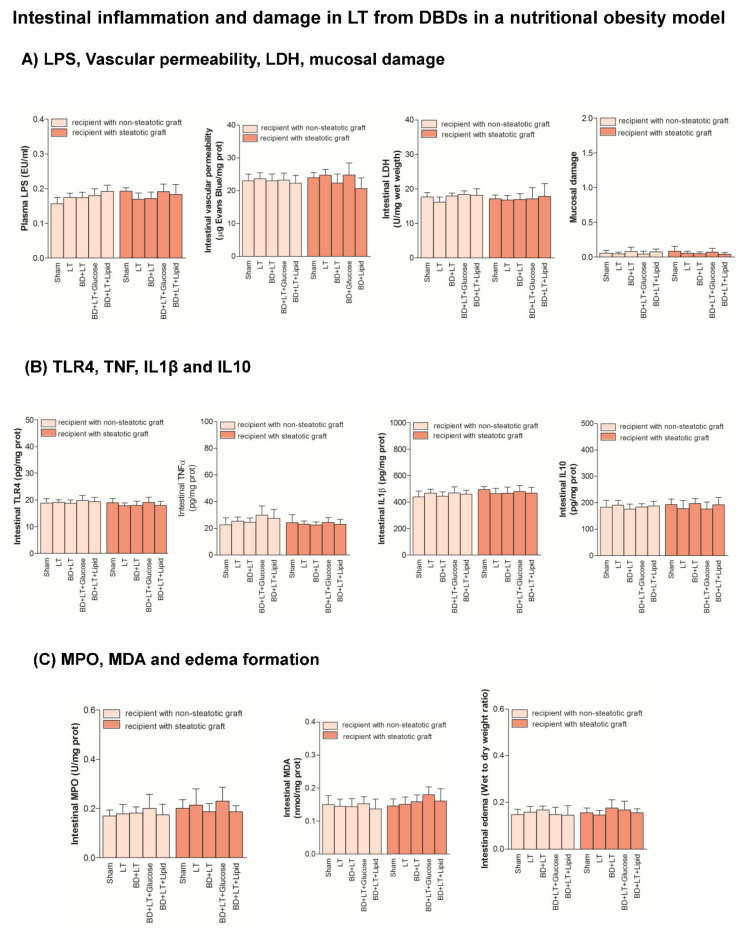
Effects of glucose and lipids on intestinal inflammation and damage in steatotic and non-steatotic LT in a nutritional obesity model. (**A**) LPS in plasma and vascular permeability, Lactate dehydrogenase (LDH) and mucosal damage in intestine. (**B**) Toll like Receptor 4 (TLR4), Tumor necrosis factor (TNF), Interleukin (IL)1β and Interleukin (IL)10 in intestine. (**C**) Myeloperoxidaase (MPO), Malondialdeyde MDA and edema formation in intestine. Sham vs. LT or BD + LT, *p* = not significant; LT vs. BD + LT, *p* = not significant. DBD, Donors after Brain death.

Our results indicated that the inflammation and damage in intestine and the hepatic damage in both liver types in a nutritional obesity model followed a similar pattern to those described in genetic obesity experimental model (Zucker rats) (Figure 5, Figure 6, Figure 7 and Figure 8). Thus, in the nutritional obesity model, our results revealed an increase in transaminases, bilirubin, damage score, ALP, von Willebrand Factor and hyaluronic acid levels in both steatotic and non-steatotic grafts of the BD + LT group when compared with the results of the LT group (Figure 5). The administration of either glucose or lipid emulsion (BD + LT + Glucose and BD + LT + Lipid groups) did not induce change in hepatic injury in non-steatotic livers, since it did not modify parameter injury parameters, in comparison with the BD + LT group. The administration of glucose (BD + LT + Glucose group) resulted in values of transaminases, bilirubin, damage score, ALP, von Willebrand Factor and hyaluronic acid levels in steatotic livers similar to those of the BD + LT group. However, the administration of lipids (BD + LT + lipid group) protected against hepatic damage in steatotic livers, with reduced transaminases, bilirubin, damage score, ALP, von Willebrand Factor and hyaluronic acid levels when compared with the results of the BD + LT group. In the nutritionally induced obesity model, lipid emulsion but not glucose promoted an increase of ATP and glycogen hepatic levels only in steatotic BD + LT group. FFA, triglycerides and total cholesterol levels were similar in all groups. BD + LT + Lipid resulted in increased phospholipid levels only in steatotic livers when compared with the BD + LT group (Figure 6). HGF, IGF1 and VEGFA levels in non-steatotic livers were similar in BD + LT, BD + LT + Glucose and BD + LT + Lipid groups. HGF, IGF1 and VEGFA levels in steatotic livers of the BD + LT + Glucose group were similar to those of the BD + LT group. However, steatotic livers of the BD + LT + Lipid group had higher HGF, IGF1 and VEGFA levels in comparison to the BD + LT group (Figure 7).

The intestinal damage and inflammation (LPS, vascular permeability, LDH, mucosal damage, TLR4, TNF, IL1β, IL10, MPO, MDA and edema formation) were similar in Sham, LT and BD + LT groups. The administration of either glucose or lipids (BD + LT + Glucose and BD + LT + Lipid groups) resulted in damage and inflammation parameters in intestine similar to those of the Sham or BD + LT group (Figure 8).

## 4. Discussion

**Figure 9:**
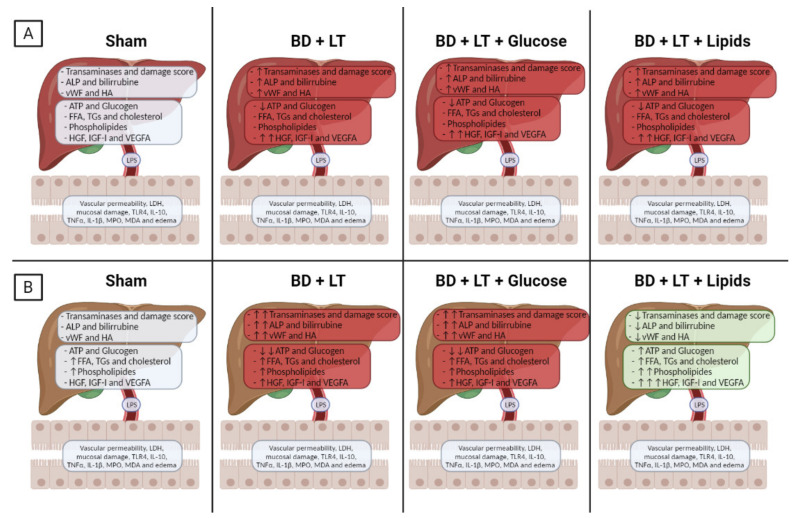
Schematic representation showing the effects of the different interventions on liver and intestine. Non-steatotic (**A**) and steatotic (**B**) livers.

To the best of our knowledge, this is the first study to report that neither treatment with glucose nor treatment with lipids protects non-steatotic livers against damage in LT from DBDs. In contrast to this, we found that lipid treatments did confer protection against hepatic damage in steatotic LT from DBDs, but this was not the case for glucose treatment (Figure 9). These results are in contrast with previous findings relating to steatotic and non-steatotic livers which were subjected to PH with or without vascular occlusion [20]. However, when assessing our findings, it should be borne in mind that the effectiveness of strategies based on the administration of glucose or lipids aimed at reducing liver damage could vary depending on the precise surgical conditions evaluated in each case (for example, warm ischemia associated with PH vs. cold ischemia associated with LT) and also depending on the type of liver that is involved (such as the presence or lack of fatty infiltration). This is the case because the underlying pathological mechanisms are very different under warm and cold ischemia conditions [40,41] and there is a dependence on the type of liver involved [27]. Here, the main finding we report is that strategies based on the use of glucose or lipids are not useful in non-steatotic LT and furthermore that treatment with lipids may be preferable to treatment with glucose as a protective strategy for steatotic livers under conditions of LT from DBDs. These results are of considerable scientific and clinical interest because they provide the opportunity to develop new pharmacological strategies for steatotic LT from DBDs that had not been reported previously.

In light of our findings, we consider that the beneficial effects of some kind of nutritional support based on a lipid treatment in steatotic livers may well be the result of lipids efficiently maintaining hepatic ATP levels, among other factors, which is crucial in order to sustain major homeostatic functions in steatotic livers after LT from DBDs. It is well known within the community that there is considerable scientific and clinical interest in interventions that are capable of preventing a drop in ATP levels in hepatic I/R processes. In the existing literature, some reports suggest that low levels of ATP after reperfusion may result in reduced tolerance of I/R injury, while in contrast to this, the application of strategies that increase hepatic ATP levels could mean that fatty livers are protected from necrosis [42].

We further aimed to understand why it is the case that lipids are a better source of energy than glucose when considering steatotic livers subjected to LT from DBDs. In this vein, it is widely accepted that when exposed to stress and a reduction in ATP levels, the liver obtains ATP primarily through fatty acid oxidation [43]. It is possible that the lipid droplets that are present in steatotic livers could provide the energy needed by the remaining hepatocytes to rebuild the liver [44,45]. If this is so, it could be the reason behind the administration of lipid emulsion potentiating ATP synthesis. Moreover, β-oxidation of the fatty acids that constitute the lipid droplets known to be present in steatotic livers could have a negative effect on the efficacious use of exogenous glucose as a means of restoring ATP levels. In fact, increasing β-oxidation of hepatic fatty acids has the effect of producing synthesis of reducing equivalents (NADH) and acetyl-coenzyme A. This in turn reduces the mitochondrial redox potential, preventing pyruvate from entering into Krebs cycle, and ultimately inhibiting glucose oxidation [46,47]. Taken together, all of this might explain, albeit partially, the maintenance of glycogen levels in steatotic livers as a result of treatment with lipids. Furthermore, we should also bear in mind that while lipid administration increased phospholipids in steatotic liver grafts, this was not the case for glucose. Phospholipids are a major cell membrane components [48,49], so lipids, in contrast to glucose, could help maintain structural integrity of cells within their microenvironment. Indeed, it is known that when some types of phospholipids split, they generate products that function as secondary messengers in signal transduction and which are also known to intervene in prostaglandin signal pathways [48,49].

Some studies have established relationships between different growth factors and certain fatty acids. Along these lines, an increase in the production of IGF-I by fatty acids has been reported in hepatopancreatic cell cultures [50]. Moreover, it has been reported that fatty acids such as linolenic and oleic acids increase the levels of IGF-1 and VEGFA, respectively, in different cells [51,52,53]. In addition to the beneficial effects of lipids on ATP, glycogen content and phospholipids, our experiments show that the influence of lipids on growth factors should also be taken into account. Our analysis shows that the levels of the proteins HGF, IGF1 and VEGFA were higher in steatotic livers treated with lipids than in those receiving glucose. In fact, after the glucose treatment, growth factor levels apparently remained unaltered. HGF, IGF1 and VEGFA play central roles in protecting against hepatic damage under hepatic I/R conditions [53,54,55,56,57] and some of these growth factors are being tested in clinical trials, for instance on patients with liver failure [58]. It is our view that the preclinical results we publish herein increase knowledge of the potential properties of lipid treatment in LT from DBDs in terms of regulating growth factor and thereby protecting against hepatic damage.

In human health, the role of the gastrointestinal microbiota is to maintain a dynamic balance with the host, playing both local and remote roles in important physiological processes such as inflammation [59,60]. It has been widely reported that polysaccharides are the most abundant dietary components [61]. In the course of intestinal fermentation, polysaccharides can promote the growth of certain intestinal bacteria, thus changing the profile of the intestinal microbiota [62], which can contribute to the development of intestinal diseases and consequently have a negative effect on organs such as the liver [63], which may result in a reduction in the quality of liver grafts [64]. Of scientific and clinical interest, it has been clearly demonstrated that microbiota imbalance can provoke immune alterations and potentiate proinflammatory pathways [59,60]. It has also been reported that gut dysbiosis causes alterations in numerous proinflammatory biomarkers such as TLR4, MDA, MPO, IL-1β or TNF-α [65,66,67,68,69,70]. Recent studies further demonstrate that microbiota may regulate the immune system, since microbial changes seem to affect IL-10 production (an anti-inflammatory cytokine) [71,72]. In addition, gut microbial disorders also affect intestinal function, since they can increase intestinal permeability [64]. LPS is the most important biomarker of gut microbial-related problems and the major biomarker of endotoxemia, caused by a microbiome disbalance as well as by problems with intestinal permeability [64]. Taking all of these observations into account, it seems that intestinal dysbiosis may result in intestinal inflammation and damage. We observed that, in recipients from DBDs fed dietary polysaccharides with or without nutritional support (based on the administration of either glucose or lipids), there were no changes in intestinal inflammation and damage. Indeed, all the parameters that are habitually altered by disbalances in microbiota, namely, LPS, TLR4, MDA, MPO, Il1, TNF, IL10, vascular permeability and edema, were unchanged and remained at control levels under the surgical conditions we applied.

Furthermore, in our surgical conditions, independently of the relevant differences in the content of dietary polysaccharides in the diets of the animals (66% polysaccharides in Ln Wistar rats and Zucker rats and 25% polysaccharides in the choline-deficient diet fed to Wistar rats), we observed similar intestinal inflammation and damage in the LT and BD + LT groups. In addition, in both the Zucker and the Wistar rats, administration of either glucose or lipids induced different effects on hepatic damage (depending on the type of the liver: steatotic or non-steatotic), but without changing intestinal inflammation and damage.

Given our current results, and in contrast with different liver diseases [12,73], the gut–liver axis is not crucial in LT from DBDs using non-steatotic or steatotic livers with 4 h of cold ischemia, since independently of the changes in hepatic damage, there was no evidence of inflammation and damage to the intestine following LT. This was also the case, as mentioned above, when the treatments with either glucose or lipids were administered. Consequently, evaluation of the potential changes in gut microbiota that might explain the improvements in intestinal inflammation and hepatic damage induced by the nutritional support strategies evaluated here was not required. Indeed, in the case of potential changes in gut microbiota, such changes would be irrelevant. Of scientific and clinical interest, the results of the current study change the dogma on the crucial role of intestinal inflammation in hepatic damage to liver grafts and post-operative outcomes after LT from DBDs. Our present results (for liver grafts with severe steatosis submitted to 4 h of cold ischemia) are different from those reported previously for warm hepatic ischemia, or LT using liver grafts submitted to 8 h of cold ischemia [74]. These different results suggest that the relevance of the gut–liver axis in LT, as well as the effects of the different pharmacological strategies applied, are dependent on the type of ischemia (warm vs. cold) and the duration of cold ischemia of liver grafts.

It is the case that glucose is routinely administered to hepatic surgery patients with post-operative hypoglycemia [20]. However, the contribution glucose makes to liver damage is still unclear. Here we report that neither lipid or glucose infusion protects non-steatotic rat livers against damage. Our preclinical study shows that lipid treatment plays a different role in LT from DBDs depending on the baseline liver status (steatotic or non-steatotic) (Figure 6). Neither strategy was useful in non-steatotic liver grafts. In the presence of steatosis, we found that lipids are more effective than carbohydrates at protecting against hepatic damage in LT from DBDs. Importantly, these benefits were independent of alterations in intestinal inflammation and damage. It is therefore our opinion that the benefits induced by lipid treatment in steatotic livers could result from the direct effects of the lipids on the liver. Clearly, much research (which goes far beyond the scope of the present study) will be necessary to determine whether these experimental results can be extrapolated to clinical practice in steatotic LT from DBDs. Nevertheless, to avoid potential risks for patients, several concerns should be considered. Although some patients suffering from liver disease present severe maldigestion and malabsorption of lipids, which leads to the administration of lipids by an enteral route being contraindicated [46], intravenous administration of lipid emulsions may resolve this issue. However, before its clinical use in steatotic LT from DBDs, some considerations need to be taken into account. It is possible that a lipid cocktail could increase free fatty acids, which are known to have hepatotoxic effects [75]. Consequently, this could lead to death in infant patients due to reduced protein lipase levels, which affects the clearance of intravenous lipid emulsion and thus increases plasma free fatty acid levels. However, we should emphasize that this chain of events is primarily associated with dosage and long-term administration of lipid emulsions [20,76]. Thus it is necessary to monitor lipid concentrations closely in those patients receiving an intravenous lipid emulsion who are at a high risk for hypertriglyceridemia. If these precautions are borne in mind by clinicians, they should be able to substantially reduce the risk of adverse effects in patients who are administered a lipid emulsion intravenously.

## 5. Conclusions

Lipid treatment is an appropriated nutritional support to protect against hepatic damage in steatotic LT from DBDs, and these benefits were independent of intestinal alterations in the recipients such findings could have the potential of improve postsurgical outcomes of steatotic liver grafts from DBD, which actually are discarded for transplantation.

Thus, could contribute to have more grafts available for LT, and consequently to reduce waiting list. Undoubtedly, more research will be required to determine if results of the present research could be applied in clinical practice.

## Data Availability

The data presented in this study are available on request from the corresponding author.
